# Improved Recursive DV-Hop Localization Algorithm with RSSI Measurement for Wireless Sensor Networks

**DOI:** 10.3390/s21124152

**Published:** 2021-06-17

**Authors:** Sana Messous, Hend Liouane, Omar Cheikhrouhou, Habib Hamam

**Affiliations:** 1 Research Laboratory of Automatic Signal and Image Processing (LARATSI), National Engineering School of Monastir (ENIM), 5019 Monastir, Department of Electrical Engineering, University of Monastir, Monastir, Tunisia; sana.messous@gmail.com (S.M.); Hend.liouane@issatkr.rnu.tn (H.L.); 2 Department of Computer Science, College of Computers and Information Technology, Taif University, P.O. Box 11099, Taif 21944, Saudi Arabia; 3 Electrical Engineering, Faculty of Engineering, Moncton University, Moncton, NB E1A3E9, Canada; Habib.Hamam@umoncton.ca; 4 Research Department, International Institute of Technology (IIT), 1.5 Mharsa Route, Sfax, Tunisia; 5 School of Electrical Engineering, Depatrment of Electrical and Electronic Engineering Science, University of Johannesburg, Johannesburg 2006, South Africa

**Keywords:** localization, multi-hop algorithms, localization accuracy, DV-Hop, RSSI, online sequential computation

## Abstract

As localization represents the main backbone of several wireless sensor networks applications, several localization algorithms have been proposed in the literature. There is a growing interest in the multi-hop localization algorithms as they permit the localization of sensor nodes even if they are several hops away from anchor nodes. One of the most famous localization algorithms is the Distance Vector Hop (DV-Hop). Aiming to minimize the large localization error in the original DV-Hop algorithm, we propose an improved DV-Hop algorithm in this paper. The distance between unknown nodes and anchors is estimated using the received signal strength indication (RSSI) and the polynomial approximation. Moreover, the proposed algorithm uses a recursive computation of the localization process to improve the accuracy of position estimation. Experimental results show that the proposed localization technique minimizes the localization error and improves the localization accuracy.

## 1. Introduction

Nowadays, Wireless Sensor Networks (WSNs) are used in several applications including healthcare, home monitoring, smart transportation [1], military tracking [2], environmental supervising, indoor navigation, etc.

These applications generally provide location-aware services where the geographical positions of sensor nodes are a fundamental part of the application data [3]. Researchers pay attention to the domain of location-aware applications and context-aware computing [3].

The presence of obstacles, noise and signal fluctuation make the sensing environment complicated. Therefore, huge challenges have emerged for localization investigation. The common solution to determine an accurate and very precise geographical location of sensor node is the Global Positioning System (GPS). That said, this localization solution is not practicable due to its high cost, power consumption, and ineffective performance in such sensor applications. Due to the disadvantages of GPS solutions, researchers proposed some alternatives to this localization problem, where a few sensor nodes are only equipped with a GPS module and a pretty energy resource to participate in the localization process of other nodes. Sensor nodes that are equipped with GPS are conceived as anchor nodes, and the other nodes of the network as unknown nodes [4].

The existing localization techniques can be broadly classified into two categories: range-based and range-free algorithms. The latter are based on network connectivity with no need of any additional hardware equipment, making them more useful in terms of low cost and energy conservation. The commonly used range-free algorithms include DV-hop [5,6], centroid algorithm [7], APIT [8], MDS-MAP [9,10] and Amorphous [11]. The range-based algorithms are based on measurement of signal characteristics, which requires expensive equipment to compute some metrics such as the angle or distance between a sensor node and its neighboring nodes [12]. The well-known range-based methods of localization in WSN are time difference of arrival [13], time of arrival [14], angle of arrival [5], and Received Signal Strength Indicators (RSSI) [15], which attracted great attention [16,17,18]. RSSI-based localization techniques have gained interest thanks to the availability of RSSI in many current technologies such as Bluetooth, Zigbee and WiFi. Since there is no need for additional hardware and due to the easy implementation, RSSI-based localization techniques are the most suitable and efficient in terms of cost and  simplicity [19]. They also ensure more accuracy in position estimation than range-free algorithms.

Moreover, in some WSN scenarios, the lack of anchors might impede the localization process. To overcome this limitation, multi-hop localization techniques can be used [20]. Indeed, in multi-hop localization, sensor nodes might use an anchor even if it is not in its communication range (not a neighbor). One of the most famous multi-hop localization algorithms is DV-Hop [6]. Although DV-Hop is simple, its accuracy is low, and therefore several improvements are proposed in the literature [3,4,21]. However, the accuracy of existing solutions is still unsatisfactory.

Aiming to enhance the localization accuracy, we designed a novel method assessing the distance between an unknown node and its neighboring anchor nodes by employing both RSSI and polynomial approximation. Our approach is an enhancement of the original DV-Hop algorithm in terms of localization accuracy and can be extended to other schemes as well. The proposed algorithm is a combination of both range-free and range-based techniques. Moreover, the proposed localization technique is based on sequential computation, which permits taking the available anchors for the localization process into consideration and, therefore, provides a better localization accuracy.

The main contributions of this study can be summarized as follows:

- We used the polynomial approximation to compute the distance between anchor and unknown nodes.
- We adjusted the estimated distance thanks to RSSI measurement.
- We introduced the recursive concept to enhance the localization accuracy of the proposed method.

The rest of this paper is organized as follows. Firstly, we expose the works related to localization in WSNs. Secondly, we describe the traditional DV-Hop algorithm, as well as its drawbacks, in Section 3. Then, our proposed localization scheme is presented in Section 4. Simulation results are given and discussed in Section 5. Finally, we conclude the paper in Section 6.

## 2. Related Works

Various range-based and range-free localization algorithms in wireless sensor networks have been proposed in the literature. The estimation of distances between the unknown node and anchor nodes can be carried out using standard approaches by various disposable techniques such as the time of arrival method [14], time difference of arrival method [13], RSSI [15], etc. Afterwards, by using the Trilateration technique, and referring to the calculated distance separating the unknown node with its neighboring anchor nodes, the geographical position of the unknown node can be estimated. It is worth noting that every unknown node should have at least three neighboring anchors to carry out the Trilateration method.

The RSSI-based localization technique is a common ranging method and is nowadays used by researchers in several localization algorithms aiming to enhance the accuracy of estimated positions of nodes. Additionally, once the majority of existing sensor nodes have the RSSI function, the RSSI-based localization technique does not require extra hardware deployment. Therefore, this method is cost-effective [22,23,24]. RSSI-based techniques could be be clustered as either fingerprinting or signal propagation modeling-based algorithms. The signal propagation modeling-based algorithms require an optimized propagation model that captures the fading and interference resulting from multi-path propagation as well as shadowing in a specified deployment area [25].

In [26], the authors propose an improved RSSI-Least Squares Support Vector Regression (RSSI-LSSVR) to optimize the position estimation accuracy as well as to minimize the cost of localization. The simulation results of this work prove that their technique can enhance the localization accuracy, minimize the localization cost and guarantee the reliability. Another RSSI-based localization technique was proposed in [27], where authors introduce a new algorithm-triangle centroid localization technique based on weighted feature points. They are interested in two positioning key issues: accuracy of RSSI value and optimization of a localization algorithm. In [28], a Received signal strength (RSS)-based algorithm to estimate positions of nodes was proposed. The authors use a Gaussian and an averaging filter to estimate distances between nodes, as well as trilateration and least square estimation for the localization process. The RSSI technique and Zigbee communication method in [29] have been also investigated to compute the distance separating the unknown node with anchor. According to the calculated distance by RSSI, less than 10 m or not, the authors calculate the position of unknown nodes by the methods of Mini-Max Positioning or Maximum Likelihood Estimation, respectively. In [30], the authors propose a novel technique of device-free human detection by using RSSI measurement in WSN based on ZigBee. Simulation results proves the validity of the proposed detection method [30].

The approach of RSSI measurement is incorporated in many improved DV-Hop algorithms, such as in [31], where the authors propose a localization technique based on RSSI and an enhanced artificial immune algorithm. In their work, they use the RSSI information admitted by the node to perform a correction coefficient in order to revise the HopCount value. Then, an enhanced artificial immune algorithm was developed to optimize the position estimation of the unknown node by Gaussian mutation in the process of a local search. In our previous work [3], we designed a new DV-Hop-based localization algorithm for large scale WSN. The proposed solution used the RSSI values for links between one-hop neighbor nodes. After being localized, nodes were promoted to act as anchors and assist the localization process with the rest of anchors.

Although several attempts have been made to enhance the DV-Hop algorithm, their localization accuracy is still unsatisfactory and did not fit context-aware applications needs. Therefore, more research efforts need to be made for this. In this paper, we propose a new localization method that leverages the polynomial approximation technique and RSSI to improve the distance estimation accuracy. In what follows, we first present an overview of the original DV-Hop algorithm and then we detail our proposed protocol.

## 3. The Original DV-Hop Localization Algorithm

In this section, we give an overview of the original DV-Hop localization algorithm and we discuss its drawbacks.

### 3.1. Unknown Node Localization Process

DV-Hop is a famous multi-hop localization algorithm. It was developed by Niculescu and Nath [5,32] and consists of three different steps, as follows.

In Step 1, known as flooding phase, each anchor broadcasts its position and a HopCount value via a beacon packet within the sensing network. The HopCount value, which is initialized to 1 and incremented in each hop, determines the number of hops between the receiving node and the sending anchor. Once this beacon packet has been received, an unknown node updates its table with the received information, $\left(x_{i},y_{i},h_{i}\right)$ from anchor *i*, where $\left(x_{i},y_{i}\right)$ is the position of the anchor *i* and $h_{i}$ is the number of hops to reach anchor *i*. If many packets from the same anchor are received by the unknown node, the minimum HopCount value is only updated.

In Step 2, after the flooding phase is completed, each anchor computes its hopSize—i.e, the average distance of hops, which is based on the distance and the minimum number of hops from other anchors. More precisely, the $HopSize_{i}$ of anchor *i* is computed according to the following equation:

(1) $$\;HopSize_{i}=\frac{\sum_{i\neq j}^{n}\sqrt{{\left(x_{i}-x_{j}\right)}^{2}+{\left(y_{i}-y_{j}\right)}^{2}}}{\sum_{i\neq j}^{n}h_{ij}}$$

where ($x_{i}$, $y_{i}$ ) and ($x_{j}$, $y_{j}$ ) are the coordinates of anchor *i* and anchor *j*, respectively, $h_{ij}$ is the minimum hop count between anchor *i* and *j*, and *n* is the total number of anchors.

Once the average distance of hops is estimated, each anchor broadcasts its HopSize to the network. This allows unknown nodes to compute their distances to reachable anchors. More precisely, an unknown node *u* estimates its distance to anchor *i* using Equation (2) as follows.

(2) $$\;d_{i}=HopSize_{i}\times h_{ui}$$

where $HopSize_{i}$ is the average distance per hop for anchor *i* and $h_{ui}$ is the minimum number of hops between node *u* and anchor *i*.

In Step 3 of the algorithm, an unknown node determines its position by Trilateration [33]. Let $d_{i}$ be the estimated distance separating the unknown node and an anchor *i*, (*x*, *y*) is the position of the unknown node, and ($x_{1}$, $y_{1}$), ($x_{2}$, $y_{2}$),…,($x_{n}$, $y_{n}$) are the coordinates of anchor nodes. The following set of equations are deduced:

(3) $$\left\{\begin{array}{l} {d_{1}}^{2}={\left(x-x_{1}\right)}^{2}+{\left(y-y_{1}\right)}^{2}\\ {d_{2}}^{2}={\left(x-x_{2}\right)}^{2}+{\left(y-y_{2}\right)}^{2}\\ .\\ .\\ .\\ {d_{n}}^{2}={\left(x-x_{n}\right)}^{2}+{\left(y-y_{n}\right)}^{2} \end{array}\right.$$

Then, Equation (3) can be transformed by:

(4) AX=B

We define the terms *A*, *X*, and *B* as follows:

$$X=\left(\begin{array}{c} x\\ y \end{array}\right)$$

$$A=2\times \left(\begin{array}{cc} x_{1}-x_{n} & y_{1}-y_{n}\\ x_{2}-x_{n} & y_{2}-y_{n}\\ .\\ .\\ .\\ x_{n-1}-x_{n} & y_{n-1}-y_{n} \end{array}\right)$$

$$B=\left(\begin{array}{c} x_{1}^{2}+y_{1}^{2}-x_{n}^{2}-y_{n}^{2}+d_{n}^{2}-d_{1}^{2}\\ x_{2}^{2}+y_{2}^{2}-x_{n}^{2}-y_{n}^{2}+d_{n}^{2}-d_{2}^{2}\\ .\\ .\\ .\\ x_{n-1}^{2}+y_{n-1}^{2}-x_{n}^{2}-y_{n}^{2}+d_{n}^{2}-d_{\left(n-1\right)}^{2} \end{array}\right)$$

By solving Equation (4) and using the least squares method [33], the unknown node can compute its coordinates as follows (Equation (5)):

(5) $$\;X={\left(A^{T}A\right)}^{-1}A^{T}B$$

### 3.2. Error Analysis of the Original DV-Hop Localization Algorithm

The original DV-Hop algorithm helps the unknown node obtain the average distance of hop (hopSize) and the HopCount value of anchors through flooding exchange. Then, by using the received information, the unknown node estimates its position. Therefore, the localization accuracy depends on the accuracy of the estimated average distance per hop. However, the estimated average distance per hop can be erroneous and thus lead to an erroneous estimated position of an unknown node.

**Figure 1:**
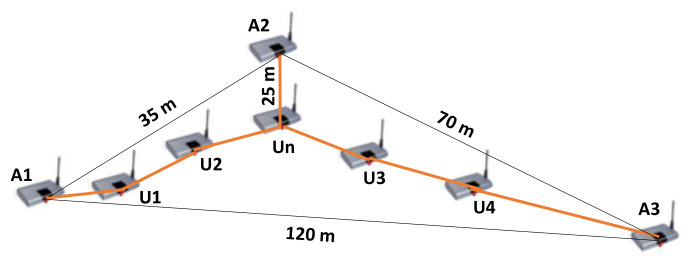
Wireless sensor network topology sample.

To illustrate the impact of average distance per hop on distance estimation between anchors and unknown nodes, we used the network topology shown in Figure 1. In this example, A1, A2, and A3 represent anchor nodes, and $U_{1},U_{2},U_{3},U_{4}$ and $U_{n}$ represent unknown nodes. The two nodes connected by an orange line can communicate directly. The average hop-distance (hopSize) of anchor A2 can be computed according to the original DV-Hop algorithm as follows: (70+35)/(4+4)=13.125. Then, the estimated distance between anchor A2 and unknown node $U_{n}$, calculated using the DV-Hop algorithm, is 13.125×1=13.125 m. However, in the graph, we can observe that the real distance between unknown node $U_{n}$ and anchor node A2 is 25 m. Therefore, the computed distance and the real distance are not closer. In conclusion, the use of the hopSize of anchors may cause errors when estimating the distance between unknown nodes and anchors.

Various improvements of the original DV-hop algorithm have been proposed [3,21,34]. Most of these techniques are based on hopSize. However, as previously proved, the hopSize computation might involve an error that consequently decreases the localization accuracy.

In this work, we aim at improving the DV-Hop algorithm to localize the unknown nodes. The proposed approach focuses on the RSSI of each link between the nodes and a polynomial approximation to determine the distance between the anchor and unknown node.

## 4. The Proposed Recursive Localization Algorithm

In this section, we design an improvement to the original DV-Hop algorithm in order to increase the localization accuracy. We aim at developing a new technique to estimate the distance between sensor nodes with more precision. The proposed technique is based on the polynomial approximation and the RSSI measurement to estimate the distance separating unknown node and their neighboring anchor nodes in the network.

### 4.1. RSSI-Based Distance Estimation

The transmitter and the receiver communicate within a distance *d*. The power strength of the radio signal broadcasted by the transmitter decreases over the distance. Therefore, the closer the receiver is to the transmitter, the stronger the signal is. Hence, the received signal strength becomes a relevant distance indicator for wireless sensor nodes. This RSSI becomes one of the more practical and effective ranging methods for wireless sensor fields thanks to its hardware cost and power consumption conservation (i.e., it does not require additional equipment) [35]. Researchers generated models for the radio channel, such as the log-normal shadowing model presented in [36]. This model describes the fading behaviour of signal propagation. The received signal power of sensor node RSS(d), which is a distance of *d* from the transmitter, is defined as in [37].

(6) $$RSS\left(d\right)\left(dbm\right)=P_{t}-P_{loss}\left(d_{0}\right)-10\tau log_{10}\frac{d}{d_{0}}+X_{\sigma }$$

where $d_{0}$ represents the reference distance, $P_{t}$ is the power of the transmitted signal, $P_{loss}$ denotes the signal power loss through the reference distance $d_{0}$, τ is the path loss exponent where its value depends on the propagation medium. $X_{\sigma }$ is the noise that is assumed to be a Gaussian random variable with a mean value equal to zero, and a standard deviation σ.

**Figure 2:**
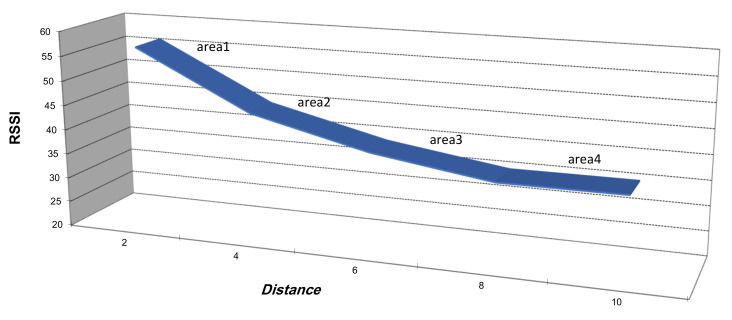
RSSI vs. distance variation.

In the experimental studies of [3,38], the authors affirmed that due to the relation between RSSI, as expressed in Equation (6), and distance (as seen in Figure 2) under various conditions and despite the promising hypothetical advantages of RSSI, even under ideal conditions, it cannot give good precision when determining internodal distances in WSN. In Figure 2, ΔRSSI and Δd represent the variation of RSSI and distance, respectively. This variation gives four different areas, as presented in plot of Figure 2. These four areas are as follows:

- Area 1: a very small variation in Δd generates a small variation ΔRSSI, and so a very small estimation error is achieved;
- Area 2: a small variation in Δd generates a small variation ΔRSSI, and so a small estimation error is achieved;
- Area 3: a very large variation in Δd generates a small variation ΔRSSI, and so a very large estimation error is achieved;
- Area 4: a very large variation in Δd generates a very small variation ΔRSSI, and so a very large estimation error is achieved.

We deduced that area 1 and area 2 are the optimal cases in terms of localization accuracy. In addition to the distance impact, the authors in [39] prove that the main drawback of the RSSI technique is its sensitivity to harsh and uncertain sensing environments. Additionally, the RSSI level changes according to the geometrical orientation of sensors and environmental characteristics, which leads to significant estimation errors. Starting from these statements, and aiming to overcome these problems, we suggested to find a combination between hop count and RSSI in order to minimize the positioning error. We used the LogNormal Shadowing Model in this work [36].

### 4.2. Proposed Localization Method

To overcome the limitation of the original DV-Hop algorithm, we aimed at developing a new technique to estimate the distance between sensor nodes with more precision. This technique replaces the use of the average distance per hop (HopSize) computed in the second step of the original DV-Hop, which might introduce localization error. In our proposed method, an unknown node will first estimate the distance to three anchors and then estimate its position recursively.

As part of our proposal, we are interested in stage 2 of the original DV-Hop and suggest a new formulation to estimate the distance between anchor nodes and an unknown node. This new formulation is a function of HopCounts and RSSI values.

In our previous work [4], the distance separating anchor node and unknown node were estimated using only polynomial approximation. More precisely, the estimated distance is equal to:

(7) $$d\left(h,\theta \right)=\sum_{k=0}^{m}\theta_{k}h^{k}$$

where θ is a vector containing *m* parameters (*m* is the order of the polynomial), and *h* is the HopCount between the unknown node and anchor node.

In this paper, we propose to use both the RSSI technique and the polynomial approximation for better precision of the estimated distance separating the unknown node and anchor node. Moreover, we considered a polynomial of order m=2. Then, by introducing the RSSI values, the estimation of the distance between anchor *i* and unknown node *j* can be computed within the following formula:

(8) $$d_{i,j}=\theta_{0}+\theta_{1}\left(h_{ij}-1\right)+\theta_{2}{\left(h_{ij}-1\right)}^{2}+DIST_{RSSI}\left(k,j\right)$$

where $\theta_{0}$, $\theta_{1}$ and $\theta_{2}$ are the polynomial coefficients. $DIST_{RSSI}\left(k,j\right)$ is the value of the estimated distance between unknown node *j* and its neighbor node *k*, which is the last node in the path from *i* to *j*. This distance is computed using the RSSI value. $h_{i,j}$ presents the minimum HopCounts between anchor *i* and unknown node *j*. In this equation, we used $\left(h_{i,j}-1\right)$ instead of $h_{i,j}$, as for the last hop, the distance was measured through the RSSI method. The aim of the polynomial approximation is to adjust the polynomial coefficients $\theta_{k}$, k={0,1,2}, so that the error introduced in the computed distance is reduced. The Polynomial coefficients $\theta_{k}$ are determined by the least squares method as follows:

(9) $$\theta_{k}={\left(H^{T}H+\lambda I\right)}^{-1}H^{T}D$$

where *H* and *D* are the matrix containing the minimal HopCount and the distance values between anchors, respectively. The coefficient λ is a regularization parameter of the least square solution, and *I* is the identity matrix.

**Figure 3:**
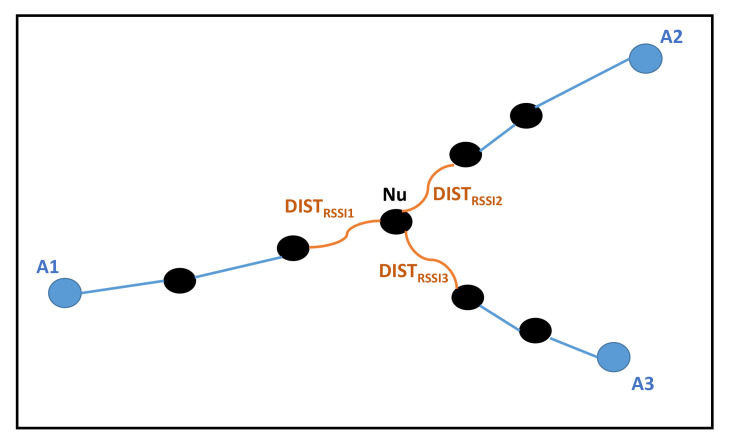
Example of a wireless sensor network. Localization of node $N_{U}$ using RSSI values and HopCount to anchors.

Figure 3 gives an example of the proposed method. Nodes A1, A2 and A3 are the anchors, and Nu is the unknown node to be localized. To compute its position, the unknown node Nu uses the RSSI values of its neighbors as well as the number of hops away from the three anchors. Then, the following equations are applied as follows:

(10) $$\left\{\begin{array}{c} d_{A1,Nu}=\theta_{0}+\theta_{1}\left(h_{1u}-1\right)+\theta_{2}{\left(h_{1u}-1\right)}^{2}+DIST_{RSSI_{1}}\\ d_{A2,Nu}=\theta_{0}+\theta_{1}\left(h_{2u}-1\right)+\theta_{2}{\left(h_{2u}-1\right)}^{2}+DIST_{RSSI_{2}}\\ d_{A3,Nu}=\theta_{0}+\theta_{1}\left(h_{3u}-1\right)+\theta_{3}{\left(h_{3u}-1\right)}^{2}+DIST_{RSSI_{3}} \end{array}\right.$$

where $h_{1u}$, $h_{2u}$ and $h_{3u}$ are the total number of hops between anchors A1, A2 and A3 and unknown node Nu, respectively.

Moreover, as sensor nodes have constrained energy and lifetime limits, they may break down for lack of energy, which leads to progressive network deconstruction and consequently the localization process will be perturbed [40]. To deal with this problem and to improve localization accuracy, we proposed, in a previous work [4], a recursive method for the localization process. Then, we used the recursive least squares estimation to derive $X_{k+1}$ as an update of the estimated position $X_{k}$.

(11) $$\;\left[\begin{array}{c} A_{k}\\ A_{k+1} \end{array}\right]X=\left[\begin{array}{c} B_{k}\\ B_{k+1} \end{array}\right]$$

The proposed algorithm recursively estimates the position of unknown nodes by improving the accuracy of the estimated result in each iteration. The recursive formula of estimated position $X_{k+1}$ of unknown node is as follows:

(12) $$\;X_{k+1}=X_{k}+P_{k+1}A_{k+1}^{T}\left(B_{k+1}-A_{k+1}X_{k}\right)$$

where *P* is a covariance matrix of the estimate and expressed as follows:

(13) $$P_{k+1}=P_{k}-P_{k}A_{k+1}^{T}{\left(I+A_{k+1}^{T}A_{k+1}P_{k}\right)}^{-1}A_{k+1}^{T}P_{k}$$

This matrix is initialized to $P_{0}=\alpha I$, where *I* is the identity matrix, and α is a very big positive number.

The mathematical development of the obtained formula is detailed in [4].

**Figure 4:**
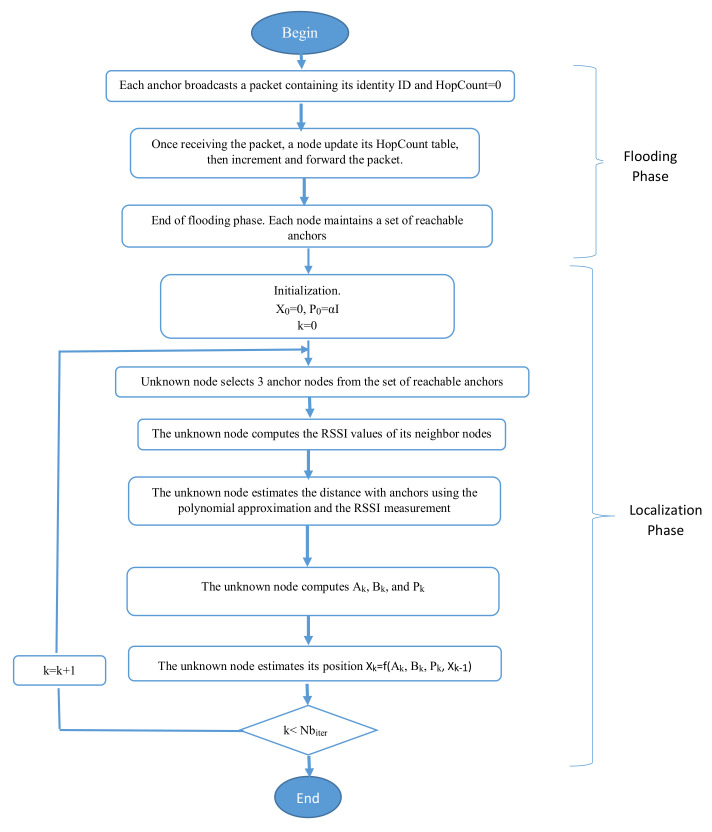
Flowchart of the proposed localization algorithm.

More precisely, the proposed algorithm is described in Figure 4 and works as follows. First, each anchor broadcasts a packet containing its identity ID and a HopCount parameter initialized to 0. The HopCount memorizes the minimal number of hops between the sending anchor and the receiving nodes. This packet will be flooded in the network and the HopCount value is incremented at each node before forwarding the packet. At the end of this flooding phase, each unknown node maintains a HopCount table containing the minimal HopCount for each reachable anchors.

Then, to estimate its position, an unknown node repeats the following five steps $Nb_{iter}$ times, where $Nb_{iter}$ is the number of iterations initially chosen. In the first step, the unknown node selects randomly three anchors from the set of reachable anchors. After that, the unknown node computes the RSSI values of its neighboring nodes (i.e., HopCount = 1). Then, the distance between unknown node and each anchor is estimated by using the polynomial approximation and the RSSI technique using Equation (8). Based on these estimated distances and using trilateration, the unknown node computes $A_{k}$ and $B_{k}$ matrix, where *k* is the iteration number. In the final step, the unknown node estimates its position $X_{k}$ by using a recursive form as in Equation (12), where this estimated position is based on the previous iteration estimated position $X_{k-1}$. The recursive form aims to improve the accuracy of the resulting position of the unknown node.

The pseudocode of the proposed localization algorithm is presented in Algorithm 1.
**Algorithm 1** Improved recursive DV-Hop algorithm.**Input:** WSN; Anchor nodes with their positions ($x_{i}$, $y_{i}$) where i=1…n, *n* is the total number of anchors;**Output:** Estimated position *X* of unknown node1: **Begin**2: $X_{0}=0$; /* initialization3: $P_{0}=\alpha *I$; /* initialize the covariance matrix *P*4: *S*= set of reachable anchor nodes, where unknown node can communicate with them5: **For** (k=0 to $Nb_{iter}$) **Do**      5.1: Selection of randomly three different anchors from *S*      5.2: Computation of RSSI values between unknown node and its neighbors      5.3: Estimation of distance between anchors and unknown node using      the polynomial approximation and the RSSI technique Equation (8)      5.4: Computation of $A_{k}$ and $B_{k}$ based on estimated distance using Trilateration, and the covariance matrix $P_{k}$ Equation (11)      5.5: Estimation of unknown node position $X_{k}$ using Recursive Least Square method Equation (12)6: **end For**;7: $X=X_{Nb_{iter}}$ /* Final estimated position of unknown node8: **end**;

## 5. Performance Evaluation

Several simulations were performed in order to evaluate the localization accuracy of the proposed localization method. The latter is compared with the original DV-Hop algorithm, Hybrid DV-Hop [3], IR-DV-Hop [4] and improved DV-Hop [41] localization algorithms through simulations by MATLAB software 2015a. For better simulation results, the reported results of all comparisons are the average over 100 trials.

### 5.1. Localization Results with Different Distributions of Anchors

**Figure 5:**
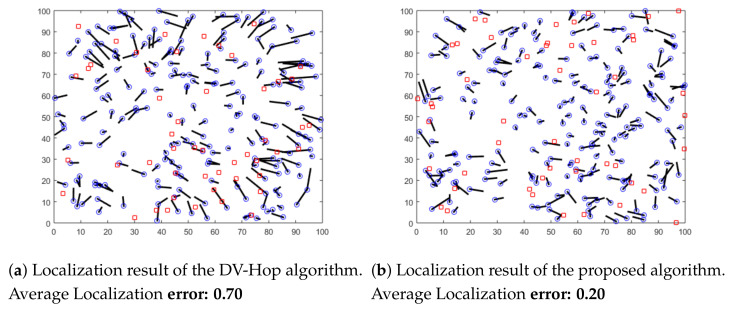
Localization results with random deployment of anchors.

**Figure 6:**
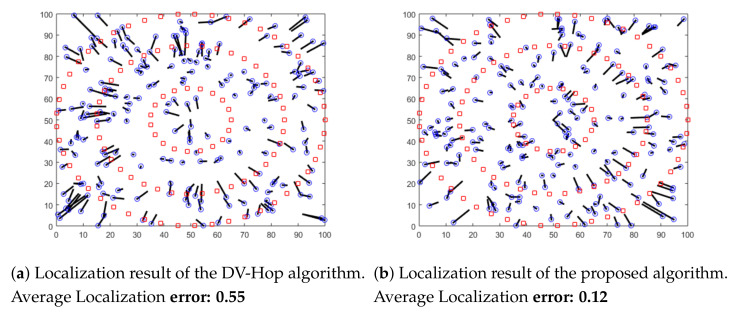
Localization results with circular deployment of anchors.

**Figure 7:**
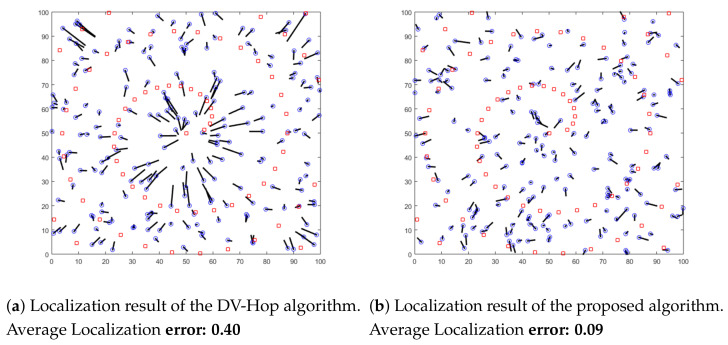
Localization results with spiral deployment of anchors.

In this section, we conduct simulations using different distributions of anchors. We aim to highlight the localization error of both the proposed algorithm and the original DV-Hop algorithm. Sensor nodes are randomly deployed in an isotropic 100 × 100 m sensing area. Nodes communicate within the same radio range of 10 m. Three different distributions of anchor nodes in the area are considered in this experiment: random, circular and spiral deployments, as shown in Figure 5, Figure 6 and Figure 7. In these Figures, anchor nodes are denoted by red squares and blue circles represent the real positions of unknown nodes, and the black line marks the localization error.

As shown in these figures, we can conclude that the localization error resulting from the proposed algorithm is lower than the error of the original DV-Hop algorithm for all different anchor placement scenarios. Therefore, our proposed solution outperforms the previous algorithms in terms of accuracy.

### 5.2. Localization Error of the Proposed Algorithm

We assumed that the anchor nodes and the unknown nodes were randomly distributed in a 100 × 100 m square monitoring field, with the communication range of the radio frequency R=10 m. Let *u* denote the number of unknown nodes in the network and $\left(x_{i},y_{i}\right)$ and $\left(\hat{x_{i}},\hat{y_{i}}\right)$ denote the real and estimated unknown node locations, respectively. In this paper, the localization criteria chosen in order to validate the proposed localization algorithm and compare it to other existent algorithms is the average localization error, which is denoted as *e* and is defined by Equation (14):

(14) $$e=\sum_{i=1}^{u}\frac{\sqrt{{\left(\hat{x_{i}}-x_{i}\right)}^{2}+{\left(\hat{y_{i}}-y_{i}\right)}^{2}}}{n}$$

**Figure 8:**
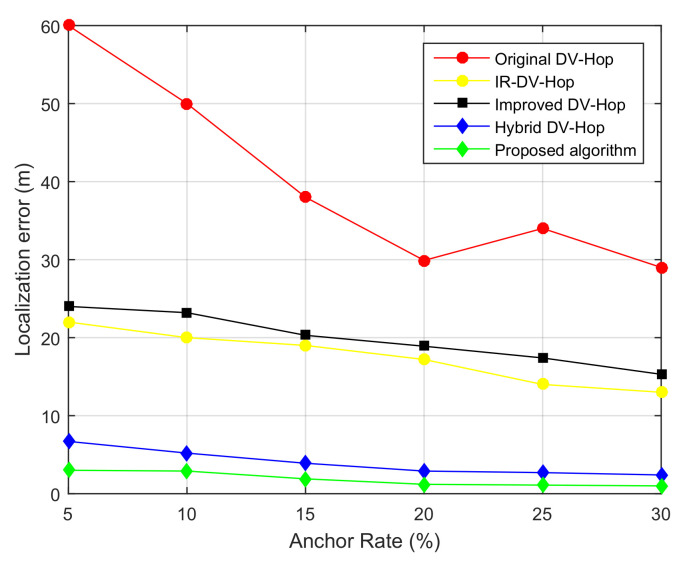
Localization error as function of anchor rate, with 100 sensor nodes and R = 30 m.

In order to emphasize the relationship between the average localization error and the ratio of anchor nodes, we conducted simulations in the Matlab 2015a environment with the following configurations: 100 sensor nodes, the ratio of anchor nodes to total nodes varies from 5% to 30%, the communication range was fixed to R=30 m. The performance comparisons are shown in Figure 8.

As seen in Figure 8 and as expected, the accuracy of localization improved with the increase in the ratio of anchor nodes in the network. In fact, the increase in the number of anchors led to maximization of the number of inputs to the Trilateration equation, used by the unknown node to estimate its distance with its neighboring anchors; thus, a better precision was achieved. It is worthy noting that, in Figure 8, the localization error decreases with the increase in the number of anchors in the network for all the compared algorithms (original DV-Hop, improved DV-Hop algorithm in [41], Hybrid DV-Hop [3], IR-DV-Hop [4], and the proposed algorithm). Furthermore, the localization error of our designed algorithm was smaller than that of its counterparts.

Table 1 shows the results of localization error when varying the anchors rate. The results are for 100 simulation runs.

**Figure 9:**
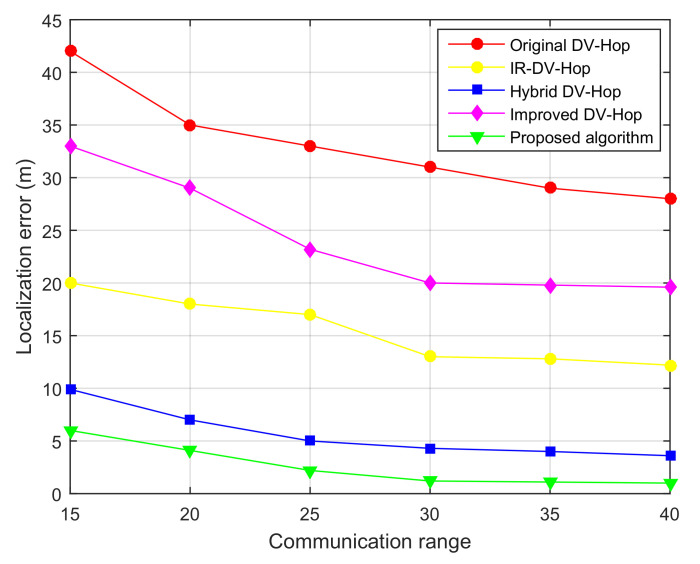
Localization error as function of transmission range, with 100 sensor nodes and ratio of anchors = 10%.

Moreover, under the same simulation parameters of 100 sensor nodes randomly deployed in the sensing field of 100×100 m, we studied the impact of the communication range on the localization error. For this purpose, we fixed the ratio of anchor nodes to 10%, and we varied the communication range *R* from 15 to 40 m. Figure 9 shows the localization error of the different compared localization algorithms.

We determined, as seen in Figure 9, the relationship between the average localization error and the nodes communication range. As is evident from this figure, the localization error decreased as the transmission radio increased. This result is expected as the increase in the transmission range led to maximization of the number of one-hop anchors and then the RSSI factor aims to improve the accuracy in the position estimation. Moreover, considering the same communication radius, the average localization error of the proposed algorithm is less than that of its counterparts.

The results of the localization error when varying the communication range for 100 simulation runs are shown in Table 2.

It is worth noting that, generally, RSSI-based localization algorithms are prone to noise effects. This can clearly be deduced from Equation (6), which shows the impact of noise in RSSI value computation. Our proposed algorithm is also vulnerable to noise effects. However, as the RSSI method is used to compute only a one-hop distance, this effect is low compared to other RSSI-based methods that use RSSI to compute the distance along the path from the unknown node to anchor. This claim is justified by the results of Figure 9, as the communication range variation has a low impact on localization error reduction.

Moreover, it is worth noting that the complexity of the proposed algorithm is O(n), where *n* is the number of iterations. The complexity used in this work is the time complexity, which is also referred to as the computational complexity. It is a quantitative measure for the amount of computer time required to run an algorithm. This amount is estimated by counting the number of elementary operations performed by the algorithm. Here, we assume that each elementary operation takes a fixed amount of time to perform.

## 6. Conclusions

Localization methods in WSN are becoming very relevant topics. They have a vast scope of applications such as monitoring human beings or objects. Considering the drawbacks of a large localization error existing in the original DV-Hop algorithm, this paper proposed an improved DV-Hop algorithm based on distance correction. Additionally, the average hop-distance of each anchor, used by the algorithm to estimate the distance between unknown nodes and anchors, is replaced by a new formula that includes received signal strength indication (RSSI) ranging technology and the polynomial approximation. The proposed algorithm has a recursive form in position calculation in order to improve the localization accuracy. Experimental results prove that the proposed algorithm minimizes the error positioning of nodes and has a higher location accuracy compared with its counterparts. 

## Figures and Tables

**Table 1:** Localization error as function of anchor rate.

Anchors Rate (%)	5	10	15	20	25	30
**Min localization error (m)**	3.995	3.715	3.214	3.725	2.353	2.216
**Max localization error (m)**	4.525	4.452	3.952	3.25	3.156	2.856
**Mean localization error (m)**	4.223	4.171	3.32	2.85	2.453	2.351
**Standard deviation**	0.018	0.020	0.035	0.015	0.045	0.022

**Table 2:** Localization error as function of communication range.

Communication Range (m)	15	20	25	30	35	40
**Min localization error (m)**	5.85	4.351	2.75	2.231	2.215	2.112
**Max localization error (m)**	6.741	5.123	3.124	3.125	3.321	3.885
**Mean localization error (m)**	6.012	4.623	2.912	2.365	2.453	2.625
**Standard deviation**	0.217	0.332	0.124	0.124	0.082	0.112

## Data Availability

Not applicable.
